# Finding and engaging patients and the public to work collaboratively on an acute infection microbiology research public panel

**DOI:** 10.1186/s40900-018-0083-5

**Published:** 2018-01-29

**Authors:** Sally Grier, David Evans, Andy Gibson, Teh Li Chin, Margaret Stoddart, Michele Kok, Richard Campbell, Val Kenny, Alasdair MacGowan

**Affiliations:** 1 Department of Infection Sciences, North Bristol NHS Trust, Southmead Hospital, Bristol, UK; 2 Department of Health and Social Sciences, University of the West of England, Bristol, UK; 3 North Bristol Microbiology Research Patient and Public Panel, Southmead Hosptial, Bristol, UK

**Keywords:** Patient and public involvement, Panel, Recruitment, Microbiology, Antimicrobial

## Abstract

**Plain English summary:**

In 2015 a microbiology team in Bristol joined a European research project that aims to develop new antibiotics to fight drug resistant infections. The microbiology team were convinced of the benefits of patient and public involvement, but had found it difficult to find former patients to work with on earlier microbiology research. This paper describes how the team overcame this challenge to successfully recruit a PPI panel to develop PPI within the European project.

The advice from people with experience in public involvement was to decide what criteria were desirable for panel membership, think about what the work of the panel might involve and how long the project will go on. The team decided that experience of suffering a serious acute infection would qualify people to comment on this project. Next, the team needed to identify ways of finding people to join the PPI panel.

The microbiology research team tried different ways to approach potential panel members. These included distributing flyers at public research events, sending emails to potentially interested people, posting a message on the hospital Facebook page and approaching eligible people known to the team. A direct approach was the most successful method – either by email, mail or in person. Ultimately 16 people were selected to form the panel. Key factors for success were planning what the work of the panel might be, perseverance despite early lack of success, and one person having overall responsibility for setting up the panel, with the support of the whole team.

**Abstract:**

**Background**

In 2015 the microbiology research team became involved in a large European programme of research aiming to bring new antimicrobial drugs onto the market to combat the increasing problem of multi-drug resistant infection. With the purpose of developing patient and public involvement (PPI) in this project, the team decided to recruit a PPI panel to work with. The microbiology team had previously worked with a PPI panel on other research, but had found it difficult to recruit members.

**Methods**

Steps taken to recruit the panel were as follows:

- Advice was sought from people experienced in co-ordinating public involvement in research.
- One person in the team had overall responsibility but the whole research team was committed and met regularly.
- Two of the team undertook training in group facilitation and connecting with the public.
- Decisions were made about the criteria for inclusion into the panel, what tasks we envisaged for the panel, the length of and frequency of meetings.
- Advertising the involvement opportunity through flyers, social media, emails and direct contact with possible panel recruits known to the research team.
- Relevant documents such as a Role Profile and expression of interest form were drafted.
- An initial public meeting was planned for all who had shown interest in the panel.
- The expression of interest form was used for us to select as broad a group as possible..

**Results**

Two out of three people who were approached directly and known by team members expressed interest in joining the panel (66%). Three out of seven members of a former panel were next (43%), then 10 out of 25 spinal infection clinic patients (40%), and finally 12 people responded to an email sent to 1261 foundation trust members (1%). No-one who was approached by indirect methods e.g. flyers or advertising on Facebook, expressed interest in the panel. Sixteen people were eventually selected for the panel.

**Conclusions**

It is possible to recruit a patient and public involvement panel for research in a discipline as challenging as microbiology. Good planning and the commitment of the research team were key to success.

## Background

Recent estimates suggest that by 2050, 10million lives per year will be at risk from multi-drug resistant bacterial infections [1]. Routine surgery will become dangerous and relatively common infections potentially life threatening. A microbiology team at North Bristol NHS Trust is involved in a European project called COMBACTE-MAGNET (Combatting bacterial resistance in Europe – Molecules against Gram-negative infections) which aims to find new ways of treating infections caused by multi-resistant Gram negative bacteria. Funding is a combination of public money from the European Union through the Innovative Medicines Initiative (IMI), and pharmaceutical companies that are interested in developing products to combat multi-drug resistant organisms [2]. The COMBACTE-MAGNET consortium is composed of 30 academic partners and five pharmaceutical companies. The microbiology team’s experience of Patient and Public Involvement (PPI) in other research prompted the team to secure some funding to develop PPI within COMBACTE-MAGNET. The microbiology team, composed of two doctors, the project manager, a nurse and an administrator, partnered with experts in public involvement at the University of the West of England to form a PPI research team and work began in January 2015. This article describes the process of setting up the PPI panel for this work.

The North Bristol Trust microbiology research team had previously worked with a PPI panel and had found this to be a positive experience. At the start PPI had been undertaken as a necessary part of obtaining funding from the National Institute for Healthcare Research (NIHR) for a Programme Grant [3]. However, the team found that the contribution of the PPI panel had improved the quality of their research. This was particularly with regard to commenting on grant applications, research design, the review of patient related documents such as consent forms and patient information sheets [4]. The panel also helped considerably by exploring consent issues relating to vulnerable groups such as intensive care patients and very frail or confused patients, which subsequently facilitated good recruitment of participants from these groups. It was also apparent that the ethics committee had taken account of the involvement of the PPI panel and this contributed to a quicker ethics review process with less necessity for amendment. However, it had been difficult to identify potential panel members at the outset and this had been achieved largely by the microbiology team approaching people they thought might be interested and willing to be involved.

INVOLVE, the NIHR Advisory Group on PPI, define public involvement in research as ‘research carried out with and by members of the public, rather than to, about or for them.’ [5] This proposes that the public are active partners in the research process at all stages from setting research agendas through to the dissemination of results. PPI should not be perceived as an intervention in research, but as an integral part of the process. PPI is well established in areas of chronic disease and mental health in the UK, but less literature has been published relating to PPI in more acute care or laboratory settings.

Early discussions with European partners in the COMBACTE-MAGNET programme have not revealed the sort of PPI in research that is now familiar in the UK. There was no PPI on the original overall project management team, which is largely based outside the UK. However, the IMI clearly has a commitment to promote the public voice in medicines development research [6]. EUPATI (European Patient Academy on Therapeutic Innovation), is a project funded by IMI which aims to make available information about medicines development, and also to teach members of the public how to be an effective voice in all stages of the research and development of new medicines [7].

In order to try to engage members of the public and patients in this new project, and in other research in the department, the research team faced the challenge of finding new panel members since the former panel had not met for a considerable time. In addition, a larger and more diverse panel, in terms of experience, age and occupational background, would potentially be better able to provide a range of perspectives on different issues and facilitate a public voice in antimicrobial medicines development research. This task was daunting and new methods of attracting the interest of potential panel members were needed.

The particular challenges associated with engaging people to work with in this area, where there is often no on-going contact with hospital services and experience of a serious infection may be a stand-alone event, prompted this article to be written about the methodology we used to contact potential public contributors. The microbiology research team, together with the PPI panel, feel that a record of the process of setting up the panel may add to the PPI body of knowledge. This is in line with the GRIPP 2 purpose of adding to the PPI evidence base by describing how patients and the public were sought, and the methods used to recruit them onto the panel [8]. Increasing the literature relating to PPI practice will serve to enhance both the quality and the impact of PPI in research. It is hoped that others who wish to implement PPI in similar areas of research to this might be able to build on our experience.

## Methods

### Planning

The microbiology research team set up regular meetings to discuss how to progress this work. One person was given overall responsibility for recruiting and co-ordinating the setting up of the panel, but the whole team were committed and contributed to the process.

It was logical that in order to establish a larger, more diverse panel some expert advice might yield promising avenues for recruitment. The PPI manager in North Bristol’s Research Department was helpful in suggesting some first steps. We consulted People in Health West of England PHWE - www.phwe.org.uk, an organisation in the South West of England that is closely linked with other health and academic organisations throughout the region. PHWE offers PPI related training for both researchers and members of the public, and also advertises involvement opportunities [9]. Two members of the team attended workshops led by PHWE to learn more about facilitating groups and working with the public. They were a good introduction into this work, and how to avoid the pitfalls of the sort of tokenistic PPI that consults with patients, perhaps out of necessity, but doesn’t take on board what they say.

All the advice suggested that careful thought about what was required would save time. Some obvious questions emerged:

- Who were we looking for – were there any particular criteria necessary for inclusion in the panel?
- What did we want them for – what did we anticipate would be the general activities of the panel?
- What time commitment did we require – how often did we plan to meet, how long were the meetings likely to be?
- How long was the project likely to run for?

The innovative nature of this particular project meant that answering these questions required lengthy discussions. It was important to the research team that the panel members had an experiential knowledge that we did not. Thus, it was decided to look for people who had experience of a serious infection requiring hospitalisation either for themselves or someone very close to them.

**Fig. 1:**
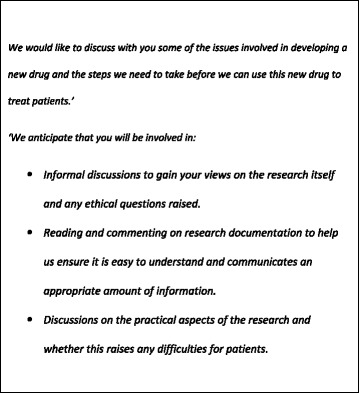
Advertisement Leaflet Wording

Anticipating the work of the panel was difficult. The hope was that as the projects developed the panel might be able to contribute to work such as commenting on patient related documents and discussing times where obtaining consent from patients for research might be difficult, such as in intensive care. However, this possibility was some way off as the projects in the programme were still in pre-clinical stages. There was also an awareness that we were attempting to develop PPI in a European project funded by a public/private partnership that was fairly unchartered territory for PPI in the UK. The wording of our advertisement reflected this uncertainty (Fig. 1: Advertisement Leaflet Wording).

We also tried to address this uncertainty when stating what time commitment would be required, saying that it was anticipated that meetings would be every two months, but that this could change over time. However, there was no expectation for panel members to commit to every meeting.

Other issues it was important to consider were advertising the involvement opportunity, the payment of expenses for panel members, drawing up a role profile and planning an initial meeting to launch the project. It was important at this stage to start to budget for the whole programme.

### Recruitment

In order to coincide with an event involving 200 members of the public who are supportive of research in the hospital a simple flyer was written advertising the opportunity, which was put inside all the information packs on the day. Two members of the team attended a regional public involvement event hosted by our local research network at which flyers were also distributed. It was decided that a colour leaflet, professionally designed and illustrated, might serve to attract potential panel members. It was given the title ‘New Drugs for Bad Bugs’, which is the strapline for COMBACTE-MAGNET. This was then put on the Research page of the hospital trust’s website, and on the Research section of the trust Facebook account.

All seven members of the former or original panel were invited to work with us again, and were sent a copy of the leaflet. The involvement opportunity was advertised through the PHWE Newsflash, a widely circulated on-line Newsletter, which included a link to the flyer. The leaflet was sent by e-mail to 1261 foundation trust members. These are people who represent the communities served by the hospital and provide feedback and support to the hospital trust on a voluntary basis. Finally, the information was sent to 25 people who had attended a spinal infection clinic and a small number of people known to the research team as fulfilling the criteria of having a serious infection that had required hospitalisation were approached.

All those who responded were invited to an initial meeting which aimed to provide more information about COMBACTE-MAGNET, and other research in the department. This was a two hour meeting, and holding this in the early evening enabled people to come on the way home from work. We had to budget for the cost of room hire and refreshments, but it was decided not to pay expenses for people attending that initial exploratory meeting.

This open event gave people an opportunity to meet the team in an informal setting, and ask as many questions as they needed to without making a commitment to continue to work with us. Indeed one person did decide not to continue involvement because it caused traumatic memories to surface, even though it was not necessary to talk about past experiences. Another advantage of the open event was that we could clarify what the work of the panel might be and what PPI means. We felt that we had been very clear that we wanted this group to work with us as research partners rather than it being a way to recruit people as trial subjects, but there were still misunderstandings.

If there had been insufficient response to methods already used to attract potential panel members other methods considered were advertising through local radio, local newspapers, contacting local G.P’s to seek people, and asking for advice about using other social media from the Hospital Trust communications office.

Panel Selection:

We had composed a role profile, so that people could see what we expected of them, and what they could expect from the research team. People were asked to fill in a short expression of interest form. This was used to ensure that the panel would have as diverse a group as possible to work with in terms of age range, experience of serious infection and educational background. Information from that initial meeting, such as the role profile, expression of interest form and advertisement leaflet, was sent to those who had expressed interest, but had been unable to attend the meeting. There was a two week deadline for the return of forms, after which the team met to discuss selection of the panel.

Criteria used for panel selection were either first-hand experience of serious acute infection that had required hospitalisation or being very close to that experience either as a close relative, partner or carer. Diversity of experience was also important, for instance we were keen to involve people who had experienced an intensive care stay as well as perhaps their partner or close relative because some of the planned studies within COMBACTE-MAGNET will recruit intensive care patients. It was decided to avoid selecting people from a single recruitment method, for instance if we had selected mainly from the spinal infection clinic patients the panel would be biased towards spinal infection. As diverse a group in terms of age, racial and social background was desirable together with ensuring that people’s motivation for joining the panel was to work with us in this project.

Ethical approval is not needed to involve the public as partners in research, because they are members of the research team, not participants or subjects of research [10].

## Results

### Planning

The commitment of the whole team and holding regular meetings kept the recruitment of the panel moving forwards. One person having overall responsibility meant that activity was co-ordinated, regular contact within the team was maintained which helped to sustain the motivation to succeed. Listening to the advice and taking the time to make decisions about what the criteria was going to be for panel membership, and what the work of the panel might involve prevented wasting time with having to rethink some aspects later on.

### Recruitment

The numbers of people seeking further information from each method of advertising is as shown below in Table 1.

**Table 1:** Recruitment method response rate

Method of Advertising	Number approached	Total Number of people seeking information	% Response of those approached
Small flyer to 200 people attending a trust event (May 15)	200	0	0
Flyer distributed at Public Involvement event (June 15)	Approximately 50	0	0
Leaflet put on to the North Bristol Trust Website research page (June 15)	Unknown	0	0
Leaflet put onto the North Bristol Trust Facebook page and link e-mailed to all research team leads (June 15)	Unknown	0	0
Advertising through PHWE Newsflash (July 15)	Total 240 (unknown how many members of the public)	0	0
Foundation trust members contacted (July 15)	1261	12	1%
7 members of the past panel contacted (July 15)	7	3	43%
Letter sent to 25 Spinal Infection Clinic patients (August 15)	25	10	40%
Other – 3 approached directly by research team (August 15)	3	2	66%

### Panel selection

Eighteen people attended the initial meeting, three of whom were close relatives of those we had approached. A further nine people who had expressed interest but were unable to attend the meeting were sent information, including a summary of the evening, copies of the slide presentations, role profile and the expression of interest form.

Ultimately the new panel was composed of 16 people. Of these we have:

- Seven women and nine men.
- Six people over 18 and under retirement age, two of whom are under 25
- There was no response from any Black or ethnic minority group. Consequently, all of the panel could be classed as ‘White British’ in terms of ethnicity.
- 2 of the panel experienced an intensive care stay during their admission
- 2 of the panel are close relatives rather than suffered a serious infection themselves.

As shown in Table 1 the biggest numerical response came via the e-mail to the foundation trust members and the letter to spinal infection clinic patients. A description of recruitment from one of each of these groups is given below. We have quoted their own words in full because each description conveys something of their motivation for joining the panel and also the type of experience that they feel they bring which has the potential to benefit the research process. The comments also illustrate the benefits to these panel members, such as satisfaction and increased knowledge:

A foundation trust member:

*I am a lay member of the North Bristol NHS Foundation Trust and I received an e-mail asking for volunteers to be a member of a Patient and Public Involvement Panel involved with research into drug resistance in bacteria and the search for new antimicrobial drugs. I have a long term interest in microbiology having spent most of my working life as a senior lecturer in environmental, industrial and agricultural microbiology and plant pathology. I felt, I might be able to make useful contributions to the discussions and other work of the panel which were outlined in the leaflet which accompanied the e-mail. Now that I am retired there was also an element of wishing to give back something to the branch of science that had given me a rewarding job for several decades of my life.*

*I also fulfilled another criterion that the leaflet said the researchers were interested in. I had had a series of urinary tract infections over several years and these had not always responded to the first antibiotics prescribed. One particularly bad infection did not respond to the first two antibiotics and resulted in emergency admission to hospital. By this stage I had been diagnosed with Parkinson’s disease and some of the possible antibiotics interacted adversely with some of my Parkinson’s drugs. I did therefore have personal experience of antibiotic drug resistance and the knock-on effects of this on the treatment of other medical conditions.*

*Sitting on the panel is an interesting experience and one I would recommend to others for its own sake. I was surprised to be offered travel expenses and a small payment for my time: I would attend anyway without this but it is a nice gesture. The panel seems to include a good range of people with differing backgrounds and experience, though how representative of the general public/patient population it is I cannot judge.* (RC).

The spinal infection clinic:

*I once boasted I’d never been admitted to hospital apart from having my family. These words were to haunt me when following two separate spinal procedures I developed sepsis. I recall nothing of falling ill, admission to A&E or lengthy spells in ICU. Countless ensuing weeks were spent recovering on a ward whilst a suitable antibiotic was sourced that worked for me. Following my discharge from hospital, my progress was monitored as the infection persisted, albeit in a weaker form. During one follow-up appointment I was asked if I would be interested as a patient, in potentially airing my views along with others, on a panel being established in respect of research into a ‘New Drugs for Bad Bugs’ three years European Study. This would tackle the increasing problem of strains of bacteria becoming progressively resistant to antibiotics. I read the literature and leaflets sent to me with great interest, it briefly outlined their research, patient participation and the content of the proposed meetings. My first reaction was yes! Followed by “I’m going to be out of my depth!” However such was my need to thank those who’d saved my life, along with finding out more about the drugs I’d been prescribed and ongoing studies, I was grateful to be given the opportunity.*

*At the introductory meeting I was made most welcome by the rest of the Microbiology Team and met others who had experiences similar to mine. However I left that initial session so full of facts and figures, I questioned if this was for me? But, subsequent meetings have opened up a new world and train of thought. I’d never previously considered the implications of the way antibiotics have previously been so liberally prescribed, or the research into fighting ‘bad bugs’. It’s fascinating, the importance of which has conveyed a deeper understanding of what exactly happened to me, and will continue to affect others until this vital study can develop new treatments. I’m one of the less vocal participants during the meetings but I sincerely hope that any contribution I make as new topics open up will go a tiny way in helping this crucial research.* (VK).

Comments from other panel members about their motivation for joining the panel related to themes of concern with the impact of antimicrobial resistance in wider society, a sense of wanting to give something back, and feeling as if they had something to offer.

- ‘*Antibiotics have played a huge part in my recovery and continuing health. Any kind of research to help improve antibiotics is of paramount importance to the future.’*
- *‘I believe I could make a useful contribution and am interested in the issues involved. Also, now that I am in the 3* ^*rd*^ *age and have free time I would like to use it constructively.’*
- *‘To help get the better of infection.’*
- *‘Concern about antibiotic resistance and interested in ethical issues in medicine, particularly in connection with commercial involvement in drug development.’*
- *‘Contribute to making a difference.’*
- *‘Interested in the subject and keen to give something back. Also to make research more relevant to patients.’*

It was felt that a panel of 16 was large enough to ensure that there would be enough people for a good discussion at times when some people were not able to attend a meeting. Only a small number that completed the Expression of Interest form were not selected, the reasons being that their experience did not fit within the criteria, or that they were looking for support for their illness, and the panel could not fulfil that role.

## Discussion

The research team was successful in recruiting a number of people to work with on the PPI panel. This was time consuming and required the commitment of the whole team to keep motivated despite the lack of success early on. The microbiology team’s belief in the benefits of PPI, plus the accountability to the project having secured some funding for PPI were important in persevering in order to recruit. One person was responsible for co-ordinating activity, communication and keeping the momentum going which also contributed to maintaining focus. Potential panel members also needed a named contact to approach to ask questions.

The NIHR advisory group INVOLVE do advise ways to find and recruit people to PPI panels, for example through local organisations, asking patients, using social media, talking with other professionals about how to access people, and they also suggest that this might take longer than anticipated [11]. Asking patients how to access potential panel members was not considered by the team, perhaps because there had been no contact with the former panel for some time and the person with primary responsibility had not been involved with the previous panel. Also, microbiologists do not have the same level of patient contact as other hospital physicians. In retrospect this could have been a good way of exploring ways to recruit to the panel. When two of the team attended training events, we did discuss recruitment issues with attendees, some of whom were members of the public, one person was specifically linked with the Somali community, but no new ideas for alternative methods of recruitment were suggested.

The authors were only able to find a very small amount in the published literature regarding researcher experience of finding people to involve and establishing a PPI panel – this was not a unique experience [12]. This is perhaps because much of the published literature on PPI in healthcare relates to areas of chronic disease and mental health. In these areas not only are there established patient organisations, but patients are more likely to have regular contact with health services. Consequently potential public contributors are not difficult to contact. Also, people with long term conditions have, over a longer period of time, developed a good deal of knowledge about their disease and experience of symptoms and the side effects of drugs and treatment. This potentially makes them more likely to seek involvement in research as they have a vested interest and an expertise that is needed to influence research agendas. People who have been hospitalised with serious infections frequently do not have follow up outpatient care post discharge from hospital. They may have an isolated hospital admission, during which they feel extremely unwell. If their infection necessitated time spent in the intensive care unit, they may not even remember it, and if they do, they, and their families may have been quite traumatised by the experience.

It could be argued that everyone in society is at risk of a serious infection, however the microbiology research team had decided to seek people who had either direct or indirect experience of being in hospital due to a serious infection. This was important as we view the panel as having a form of experiential knowledge that the microbiology research team hasn’t got. Almost all of the panel have been hospitalised at some time with infection, some have only had one such encounter with health services, others have had repeated admissions with a long-term infection. Two of the panel had an intensive care stay, and the husband of one of those is also on the panel. In our opinion individuals with this experience are best placed to comment on research that might include participants who are very sick and perhaps unable to consent for themselves. Our discussions with them have already indicated that not only do they have concern for the wider implications of antimicrobial resistance, but also their own experience of their illness, and treatments gives them insight that they would not otherwise have, for instance finding that their infection is resistant to the prescribed antibiotic, or suffering side effects associated with prescribed antibiotics. When asked directly if they felt it important that they had first-hand experience of a serious infection the consensus was that they did.

Hardavella et al. suggest that finding patients to participate in research as research subjects is difficult if the research is laboratory based, and perhaps finding people to become involved in PPI in laboratory based research is even more difficult [13]. It is clear from the number of respondents in Table 1 that personal contacts within the research team yielded a much higher response rate than advertising more widely. It also seems that a link with the trust through being a foundation trust member prompted more expressions of interest than through wider advertising. Without exception all respondents had been approached directly either by e-mail, letter or in person. There is evidence to suggest that a good relationship between the research team and the PPI panel has a positive influence on the quality of the PPI contribution to the research [14]. It could be that a good experience of contact with members of the research team, and with the hospital for the foundation trust members prompted people to respond. In fact in our case wider advertising didn’t result in any interest, despite the prominence of the problems of antibiotic resistance in the media. One can only speculate why that was, but we were fortunate that there was more than enough interest to form our panel. We had planned to advertise through local press and GP surgeries if necessary, but ultimately this was not needed.

The question of how many people are needed for a PPI panel is not clear. It is logical that enough people to generate a good discussion is required, and that they may gain confidence from each other. A very large group might work against good and open discussion, perhaps by inhibiting some panel members from contributing. It is unlikely that everyone can attend every meeting, so this was also bourne in mind. The results section indicated that there was more interest than needed, and the expression of interest form was used to ensure as broad a panel in terms of experience and age as possible. The final number of 16 was selected as experience suggested that it was likely that some may drop out, which has indeed happened and we currently have a panel of 13.

The panel is a self-selected group – those who responded to our recruitment strategies are already those who are already interested in research. This perhaps means that they hold a particular bias, but it may also make them more likely to voice opinions that might contradict researchers in the interests of patients. Indeed the people approached by the microbiology research team directly for the initial panel, and for this one, were approached with the knowledge that they might do just that.

The question of how representative the panel is of the wider community has been considered by the team, and the panel, but this is not easy to address with limited resources. In many ways it is impossible to achieve a group reflecting the true diversity of the local population in terms of ethnicity and socioeconomic mix [15]. For the research team representativeness in terms of a range of experience of the sort of infections that result from Gram-negative bacteria seemed important. Bacteria that are classed as Gram-negative have a particular type of cell wall which can defend them against some antibiotics. They are the cause of most hospital acquired infections in the intensive care unit [16].

There is a good deal of awareness of the fact that the panel does not represent the diversity of the local community and also the various different European communities that the breadth of this project involves. However, a breadth of experience and a willingness to contribute is of huge importance. Involving seldom heard groups either due to ethnic background or social class is a well-known problem. A recent systematic review identified only a small number of studies outside the United States involving Black and ethnic minority members in PPI [17]. We felt that our advertising had been quite broad-reaching, and we were keen to meet anyone who expressed interest. If there was a need to seek the opinions of a particular group it may be necessary to find a way of approaching that group, and it is hoped that an awareness of the panel’s limited representativeness will prevent unhelpful assumptions from being made. It is recognised that perceptions of illness and research along with the needs of different groups amongst the population can also influence the opportunity to participate in research as well as the outcome.

Finally, it should be noted, that public involvement is not cost free. Significant time was spent in the preparation stages, and seeking some training on involving members of the public. Then there are practical costs to consider, printing, room hire and catering, and expenses for panel members. We decided not to pay expenses for the initial open meeting, partly because it was an open meeting and we weren’t sure how many were going to attend, but also because the initial meeting was a place for people to seek information and ask questions, not the beginning of the work of the panel. We did, however, have to budget for the meetings going forward. INVOLVE have published guidelines on budgeting for involvement, which we found very helpful. [18].

## Conclusions

It is possible to establish PPI to contribute to research in microbiology and antimicrobial drug development. Seeking advice at the start was of paramount importance, and then thinking carefully about who we wanted and what we wanted them for. Ways of advertising the opportunity were needed, and perseverance to keep trying when there was no initial interest. Whilst the preparation took time, it seemed to make things come together smoothly and professionally, and so was time well spent. In retrospect asking patients for ideas about how to engage with potential PPI panel members could have been an effective method to add. One person having primary responsibility and being contactable contributed to building a relationship with potential panel members quickly.

The initial meeting, which was an open event, meant that people could meet with us, and have an opportunity to investigate what was involved with no commitment to continue. Asking people to fill in a brief expression of interest form helped us to select as broad a group as possible and ensure that there was some balance in the panel in terms of experience, age and background. It also lent a formality to the process, and made people think about why they wanted to take part. This may have fostered a sense of commitment that may not otherwise have been there.

The research team have been hugely impressed by the interest and commitment of the patient panel. Whilst initially we were unsure how to establish the panel, and were discouraged by the failure of early advertisements to secure any interest, persistence, and the commitment of the whole team to PPI resulted in the successful recruitment of the panel.

## Abbreviations

COMBACTE - MAGNET: Combatting bacterial resistance in Europe - Molecules against Gram-negative infections
EUPATI: European Patient Academy on Therapeutic Innovation
IMI: Innovative Medicines Initiative
NIHR: National Institute for Healthcare Research
PHWE: People in Health West of England
PPI: Patient and Public Involvement
